# The Impact of mRNA Structure on Guide RNA Targeting in Kinetoplastid RNA Editing

**DOI:** 10.1371/journal.pone.0012235

**Published:** 2010-08-17

**Authors:** Larissa Reifur, Laura E. Yu, Jorge Cruz-Reyes, Michelle vanHartesvelt, Donna J. Koslowsky

**Affiliations:** 1 Comparative Medicine and Integrative Biology Program, College of Veterinary Medicine, Michigan State University, East Lansing, Michigan, United States of America; 2 Cell and Molecular Biology Program, College of Natural Sciences, Michigan State University, East Lansing, Michigan, United States of America; 3 Department of Biochemistry and Biophysics, Texas A&M University, College Station, Texas, United States of America; 4 Dow Corning, Teachers for a New Era, Michigan State University, East Lansing, Michigan, United States of America; 5 Department of Microbiology and Molecular Genetics, Michigan State University, East Lansing, Michigan, United States of America; Lehigh University, United States of America

## Abstract

Mitochondrial mRNA editing in *Trypanosoma brucei* requires the specific interaction of a guide RNA with its cognate mRNA. Hundreds of gRNAs are involved in the editing process, each needing to target their specific editing domain within the target message. We hypothesized that the structure surrounding the mRNA target may be a limiting factor and involved in the regulation process. In this study, we selected four mRNAs with distinct target structures and investigated how sequence and structure affected efficient gRNA targeting. Two of the mRNAs, including the ATPase subunit 6 and ND7-550 (5′ end of NADH dehydrogenase subunit 7) that have open, accessible anchor binding sites show very efficient gRNA targeting. Electrophoretic mobility shift assays indicate that the cognate gRNA for ND7-550 had 10-fold higher affinity for its mRNA than the A6 pair. Surface plasmon resonance studies indicate that the difference in affinity was due to a four-fold faster association rate. As expected, mRNAs with considerable structure surrounding the anchor binding sites were less accessible and had very low affinity for their cognate gRNAs. *In vitro* editing assays indicate that efficient pairing is crucial for gRNA directed cleavage. However, only the A6 substrate showed gRNA-directed cleavage at the correct editing site. This suggests that different gRNA/mRNA pairs may require different “sets” of accessory factors for efficient editing. By characterizing a number of different gRNA/mRNA interactions, we may be able to define a “bank” of RNA editing substrates with different putative chaperone and other co-factor requirements. This will allow the more efficient identification and characterization of transcript specific RNA editing accessory proteins.

## Introduction

One of the most striking examples of small RNA regulation of gene expression is the process of RNA editing in the mitochondria of trypanosomes [1]. In these parasites, RNA editing involves extensive uridylate insertions and deletions within most of the mitochondrial mRNAs. The editing process involves a protein based cleavage/ligation mechanism with the information for the uridylate insertions/deletions supplied by small transacting guide RNAs (gRNAs) [2]. Hundreds of gRNAs are responsible for directing the sequence changes that create start and stop codons, correct frameshifts and for many of the mRNAs, generate most of the open reading frame of the mRNA. Editing can also create alternative distinct open reading frames [3], [4]. The RNA editing process is developmentally regulated in a transcript specific manner. For example, the cytochrome b (CYb) and cytochrome oxidase II mRNAs are edited in the insect and stumpy-bloodstream form parasites and are primarily unedited in slender bloodstream forms [5], [6]. In contrast, Complex I proteins (NADH dehydrogenase subunits) are preferentially edited in bloodstream forms (for review see [7]). The small RNAs (∼60 nts) that guide the editing process are found in all developmental stages, suggesting that the regulation of RNA editing is not at the level of gRNA availability [8].

RNA editing begins with two linked processes: the recognition and assembly of a gRNA onto its cognate target, followed by the assembly of the correct editosome (dependent on the type of editing (deletional or insertional)) onto the paired substrate [9]. A key role in ensuring accurate and efficient RNA editing must be the initial gRNA - mRNA pairing event. Target selection by the gRNA is governed by the anchor sequence (4–16 nts) at the 5′ end of the molecule. The target mRNA contains an anchor-binding site (ABS) complementary to the gRNA anchor, located just 3′ to the editing domain. Extensively edited RNAs appear to be edited generally in the 3′ to 5′ direction by the sequential utilization of many gRNAs. While the initiating gRNA can begin the process by base pairing to the pre-edited mRNA, all subsequent gRNAs require editing of the target by the downstream gRNA to create the anchor-binding sequence necessary for recognition. Considering the existence of hundreds of anchor binding sites, there must be a variety of secondary and tertiary features that must be disrupted for gRNAs to bind the ABS. The sequence and structure surrounding the ABS could be influencing the nucleation event by the gRNA and also the binding affinity of this bimolecular interaction. Once the duplex between ABS and gRNA anchor forms, it should be particularly stable to allow further reorganization of the two RNAs into the core structure necessary for proper editing.

In this study, we show that the ability of the gRNA to efficiently pair with its cognate target is dependent on both the degree of secondary structure surrounding the anchor-binding site and the base composition of the targeting region. Efficient pairing however, does not guarantee *in vitro* editing at the correct editing site, suggesting that some accessory factors may be involved in RNA folding and proper presentation of the correct site to the editosome. This suggests that different gRNA/mRNA pairs may require different “sets” of accessory factors for efficient editing. By characterizing a number of different gRNA/mRNA interactions, we may be able to define a “bank” of RNA editing substrates with different putative chaperone requirements. This may allow the more efficient identification and characterization of transcript specific RNA editing accessory proteins.

## Materials and Methods

### Oligonucleotides

All oligodeoxynucleotides (ODNs) (Table 1) were purchased from Integrated DNA Technologies, Inc. (Coralville, IA). The sequence of the oligoribonucleotide ND7-550 (Dharmacon (Boulder, CO) used for the SPR experiments is as follows: 5′AAAAACAUGACUACAUGAUAAGUACAAGAGGAGACAGACGACAGUGUCCACAGCACCCGUUUCAGCACAG-3′.

**Table 1:** Oligodeoxyribonucleotides.

ODN name	Sequence (5′ to 3′)
**gRNA**	
T7-22	AATTTAATACGACTCACTATAG
gA6-14	AAAAAAAAAAAAAAATAATTATCATATCACTGTCAAAATCTGATTCGTTATCGGAGTTATAGCCCTATAGTGAGTCGTATTAAATT
gND7-550	AAAAAAAAAAAAAAATATTCACATTTATATCATCTTACACTTAATCCACTGCATCCCTATAGTGAGTCGTATTAAATT
gND7-550sU	TATTCACATTTATATCATCTTACACTTAATCCACTGCATCCCTATAGTGAGTCGTATTAAATT
gND7-506	AAAAAAAAAAAAAAATTCACTATCTACACTAACTATACTACAGGTTATTTACATCGTAGCCCTATAGTGAGTCGTATTAAATT
gCYb-558	AAAAAAAAAAAAAAATTATTCCCTTTATCACCTAGAAATTCACATTGTCTTTTAATCCCTATAGTGAGTCGTATTAAATT
**mRNA**	
ND7UHR3 - 5′ half	CATCAATAAATCTTATCCCCTCTCCTCCAACTGTGCCTATAGTGAGTCGTATTAAATT
ND7UHR3 - 3′ half	CATTGTTCTACACTTTTATATTCACATAACTTTTCTGTACCACGATGCAAATCACAAATTT
ND7UHR3 Bridge	GATAAGATTTATTGATGAAATTTGTGATTTGC
ND7UHR3 Forward	AATTTAATACGACTCACTATAG
ND7UHR3 Reverse	CATTGTTCTACACTTTTATATTCACATAAC
ND7-550 - 5′ half	AATTTAATACGACTCACTATAGGGATACAAAAAAACATG
ND7-550 - 3′ half	ACTACATGATAAGTACAAGAGGAGACAGACGACAGGTCCACAGCACCCGTTTCA
ND7-550 Bridge	TACTTATCATGTAGTCATGTTTTTTTGTATC
ND7-550 Forward	AATTTAATACGACTCACTATAGGGATACAAAAAAACATG
ND7-550 Reverse	GTGCTGAAACGGGTGCTGTGGACCTGTCGTC
T7A6 Forward	AATTTAATACGACTCACTATAGGAAAGG
A6U Reverse	TATTATTAACTTATTTGATCTTATTCTATAACTCCAA
A6P1 Reverse	mUmATTTGATCTTATTCTATAACTCCAATCACAAC
A6Ush2 Reverse	CTTATTTGATCTTATTCTATAACTCCAA
T7CYbU Forward	AATTTAATACGACTCACTATAGGGTTATAAAT
CYbU Reverse	GGCCGCTCTAGAACTAGTGG
**RNase H assay ODNs**	
A6U	CTATAACTCC
CYbU	ATTAAAAGAC
ND7UHR3	CGATGCAAATC
ND7-550	GTGCTGTGGAC
**SPR assays**	
3′BigSK-biotin	CACTAGTTCTAGAGCGGCC-biotin
A6BigskBIAbridge	GCTCTAGAACTAGTGTATTATTAACTTATTTG
ND7550BigskBIAbridge	GCTCTAGAACTAGTGCTGTGCTGAAACGGG

### Templates for RNA transcription

ATPase subunit 6 (A6) and CYb templates were PCR amplified using the forward and reverse primers listed in Table 1 and plasmids described previously [10]. Templates for ND7UHR3 and ND7-550 were generated by ligation of 1 nmol of 5′ ^32^P-labeled 3′ half to 1 nmol of the 5′ half (Table 1), using 1 nmol of the bridge ODN and 25 U of T4 DNA Ligase (Roche) in 66 mM Tris-HCl, 5 mM MgCl_2_, 5 mM DTT, 1 mM ATP, pH 7.5, at 22°C, overnight. The ligated single stranded DNA product was gel purified and then amplified using the appropriate forward and reverse primers.

### RNA transcription and radioactive 5′-end-labeling

RNAs were transcribed using the T7 RiboMax kit (Promega) according to manufacturer directions. For 5′ end-labeling, RNAs were treated with calf intestinal alkaline phosphatase (New England Biolabs) followed by labeling with 50 µCi of [γ^32^P]-ATP, using T4 Polynucleotide Kinase (Invitrogen) and standard procedures. All RNAs were gel purified on 8% (mRNA) or 15% (gRNA) polyacrylamide gels containing 8M urea. The RNAs were eluted in an RNA elution buffer (10 mM Tris pH 7.8, 0.1% SDS, 2 mM EDTA, and 0.3 M NaOAc pH 7.0) in the presence of phenol, recovered by ethanol precipitation, and quantified using a Cary 50 spectrophotometer.

### Secondary Structure Prediction

Predicted secondary structures and free energies were obtained using mfold version 2.3, http://mfold.bioinfo.rpi.edu and DINAMelt, http://dinamelt.bioinfo.rpi.edu [11], [12]. Confirmation of the predicted structures and accessibility of the ABS was obtained using ODN directed RNase H assays.

### ODN directed RNase H assays

Fifty pmoles of 5′ ^32^P-labeled mRNAs were renatured after gel-purification by heating to 70°C for 3 min and slow cooling (2°C/min) to 27°C in RH buffer (40 mM Tris-HCl pH 7.5, 100 mM KCl, 2 mM MgCl_2_, 1 mM DTT). The sample was incubated at 27°C for 30 additional min and then quenched on ice. One unit of RNase H and different concentrations of the appropriate ODN (at the molar ratios of 1∶1, 1∶5, 1∶10, and 1∶30 substrate∶ODN) were added. Aliquots were taken at 1, 15 and 30 min of incubation and the reactions stopped with addition of formamide loading buffer (80% (v/v) formamide, 10 mM EDTA, 1 mg/ml bromophenol blue, 1 mg/ml xylene cyanol). Samples were resolved on 8% 8M urea polyacrylamide gels. All assays were conducted in triplicate. The percentage of RNase H digestion (radioactive bands) was determined using a Storm PhosphorImager (Molecular Dynamics, Sunnyvale, CA) and the ImageQuant™ software for image analysis. The fraction cleaved was calculated as the signal in the band corresponding to cleaved mRNA divided by the total signal of the cleaved and free bands.

### Determination of Binding Affinity

Binding affinities were determined by electrophoretic mobility shift assays (EMSA) as previously described, except that the gRNA/mRNA hybridization time was increased to three hours [13]. gRNA concentrations were set at either 5 or 10 nM. The concentration range used for each mRNA in the reactions was as follows: **ND7-550**- 0 nM, 0.78 nM, 1.56 nM 3.13 nM, 6.25 nM, 12.5 nM, 25 nM, 50 nM, 100 nM, 200 nM; **A6U** - 0 nM, 2 nM , 4 nM , 8 nM, 12.5, 16 nM, 25 nM, 37 nM, 50 nM, 75 nM; **ND7UHR3** - 0, 20 nM, 60 nM, 145 nM, 235 nM, 470 nM, 695 nM, 765 nM, 945 nM, 1.4 µM, 1.98 µM; **CYbU** – 0, 75 nM, 125 nM, 250 nM, 500 nM, 750 nM, 960 nM, 1.51 µM, 1.97 µM, 3.0 µM. The apparent affinity constant (K_D_) of gRNA binding was extracted from data-point fitting using KaleidaGraph 3.5 and the following binding model: K_D_ = [gRNA_f_][mRNA_f_]/[complex]. Where: complex = gRNA bound to mRNA; [gRNA_free_] = [gRNA_total_]−[complex]; [mRNA_free_] = [mRNA_total_]−[complex] [14]. The K_D_ value is an average of four experiments and the standard error was calculated from the difference in these values.

### Determination of Rate Constants

The mRNA/gRNA pairs used in this study were all synthesized *in vitro* with the exception of ND7-550 that was chemically synthesized by Dharmacon Inc. The mRNAs were ligated to the 3′BigSK-biotin ODN tag by annealing the tag to the 3′ end of the appropriate mRNA using a bridging ODN [15]. The biotinylated mRNAs were gel extracted without phenol and purified using ultra-free MC membranes (Millipore) and microcon tubes (YM-50, Millipore) according to the manufacturer's directions. The gRNAs were also purified using Ultra-free MC and the YM-10 or 30 microcon tubes. The RNA samples were then diluted in running buffer (100 mM Tris pH 7.5, 0.1 mM EDTA, 2 mM MgCl_2_, and 100 mM KCl). The biotinylated mRNA was diluted to 10 nM and 50–150 resonance units (RU) of mRNA was attached at a 5 µl/min to a streptavidin coated SA sensor chip (BIACORE, Uppsala, Sweden). Two cells were immobilized with mRNA, one was left unmodified to serve as a reference cell, and one cell was immobilized with the ODN tag as a control cell. Binding studies were carried out at 27°C on a BIACORE 2000 (BIACORE, Uppsala, Sweden) running all four cells in series with respective cycles: 1) 100–300 µl gRNA injection at 5–10 µl/min (to obtain association at varying concentrations of gRNA). 2) Buffer flow (for dissociation of gRNA) 5–10 µl/min for 60–180 min. 3) Regeneration (50 µl injection of regeneration buffer (8 M Urea), two 250 µl injections of running buffer at 50 µl/min). Separate fits for each association and dissociation curves were analyzed globally from each experiment to obtain k_on_ and k_off_, individually, and the results were averaged. The dissociation equilibrium constant (K_D_) was calculated from the averages of the rate constants using the equation: 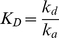. The errors reported for the rate constants were based on the variances of all curves.

### gRNA Directed Cleavage Assays

Procyclic-form mitochondria were isolated, lysed and cleared by centrifugation. The extract (∼2×10^10^ cell equivalents/ml) was then separated by glycerol gradient sedimentation as previously described [16]. We additionally used a mitochondrial extract enriched for the editing complex by Q-Sepharose chromatography, obtained from Dr. Cruz-Reyes [17], [18]. Editing complexes were further treated with 0.5 mM inorganic pyrophosphate (PPi) to inhibit the RNA ligase reaction and improve cleavage detection [19]. Cleavage assays were conducted in triplicate with glycerol fractions and purified complexes. For each cleavage reaction, approximately 0.1 pmols of 5′-^32^P-labeled mRNA (60 Kcpm) and 1 pmol of cognate gRNA were heated to 70°C (3 min), slow cooled (2°C/min) to 27°C and incubated at 27°C for 30 min. Then, 2 µl of purified editing complexes or 10 µl of the glycerol fraction were added and the reaction incubated for an additional hour in 10 mM KCl-MRB buffer (25 mM Tris-HCl pH 7.9, 10 mM MgOAc, 10 mM KCl, 1 mM EDTA, 0.5 mM DTT, 1 mM CaCl_2_, 5% glycerol). The cleavage reaction was terminated by the addition of 2 µl of stop buffer (130 mM EDTA, 2.5% SDS) followed by phenol/chloroform/isoamyl alcohol (25∶24∶1) extraction and ethanol precipitation. Samples were resolved on 8% (w/v) denaturing polyacrylamide gels. The cleavage product amount was calculated as the percentage of total input mRNA.

### Solution Structure Probing

To probe for single stranded regions in the ND7-550/gND7-550 complex, 5′-^32^P-labeled mRNA was hybridized to a 10-fold excess of gRNA in NEB buffer 2 (10 mM Tris pH 7.5, 50 mM NaCl, 10 mM MgCl_2_, and 1 mM DTT) by heating to 70°C for 3 min, slowly cooling to 27°C, and keeping at 27°C for approximately 1h. The single stranded specific Mung Bean Nuclease (NEB, Ipswich, MA) was then added (1.5 U, 3 U, or 4.5 U) and the sample was incubated at 27°C for 10 min. The reaction was phenol/chloroform/isoamyl alcohol (25∶24∶1) extracted and treated as described in the gRNA directed cleavage assays.

## Results

### Description of mRNAs and analyses of their predicted secondary structures

**Figure 1:**
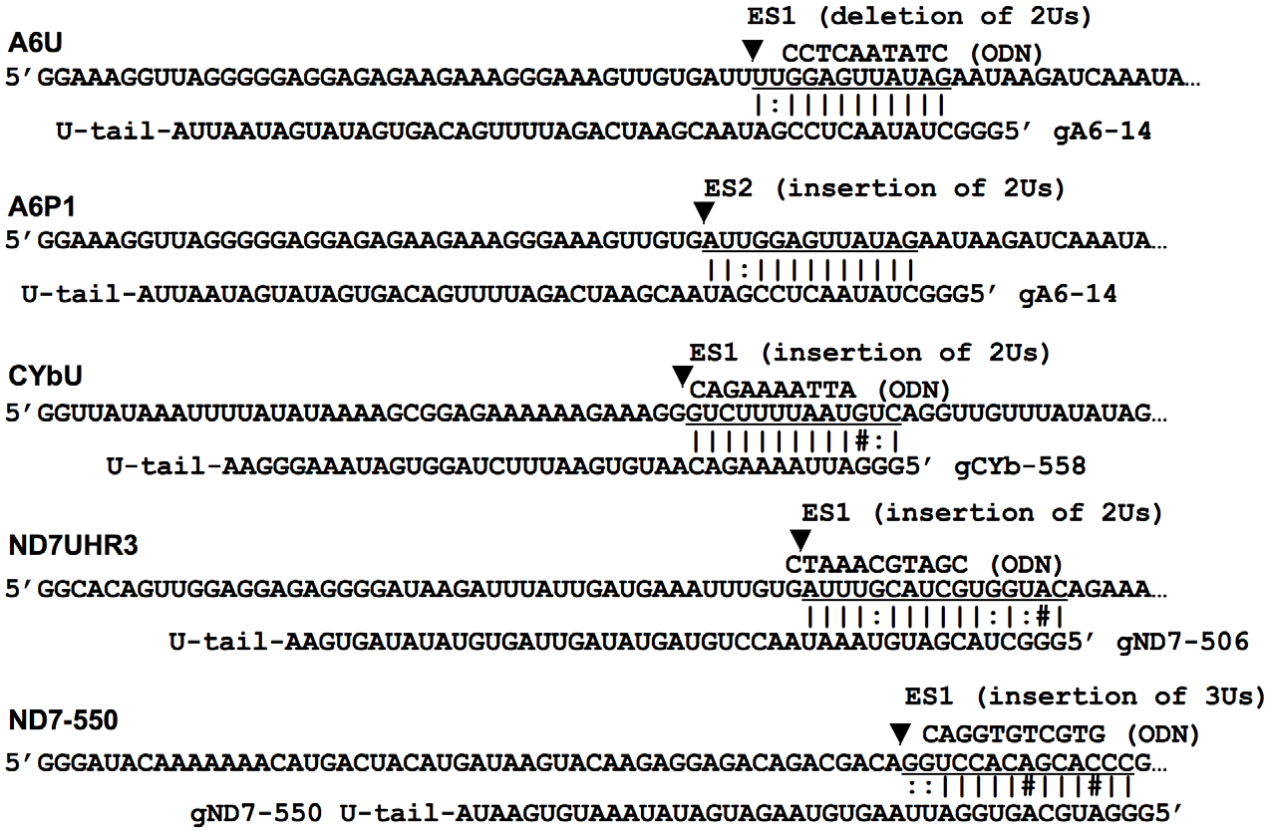
mRNAs aligned with gRNAs and ODNs. The mRNA anchor binding site (underlined) is complementary to the gRNA anchor and ODN. Watson-Crick (|), non-Watson-Crick (:) base pairs, mismatches (#) and the first editing site (ES, arrowhead) are indicated. The mRNA sequences continue at the 3′-end, as in figure 2.

Four different mRNA/gRNA pairs were used to investigate the role of sequence and structure in gRNA targeting; A6U/gA6-14, CYbU/gCYb-558, ND7UHR3/gND7-506, and ND7-550/gND7-550 (Fig. 1). Both the ATPase 6 (A6) and Cytochrome b (CYb) pairs have been previously described [10]. The two NADH dehydrogenase 7 (ND7) substrates, ND7UHR3 and ND7-550, are from two separate regions within the 5′ editing domain of the ND7 mRNA and require different gRNAs to be edited [20], [21]. ND7UHR3 contains the ABS for the 5′ domain initiating gRNA, gND7-506. ND7-550 is located near the 5′-end of ND7 and contains the ABS for gND7-550. This ABS is a span of 14 nucleotides that are not edited in the mature transcript. Like A6, the two selected regions of the ND7 mRNA are constitutively and extensively edited. In contrast, editing of CYb is developmentally regulated in that the CYb mRNA is not edited in the slender bloodstream stage of the trypanosome life cycle.

**Figure 2:**
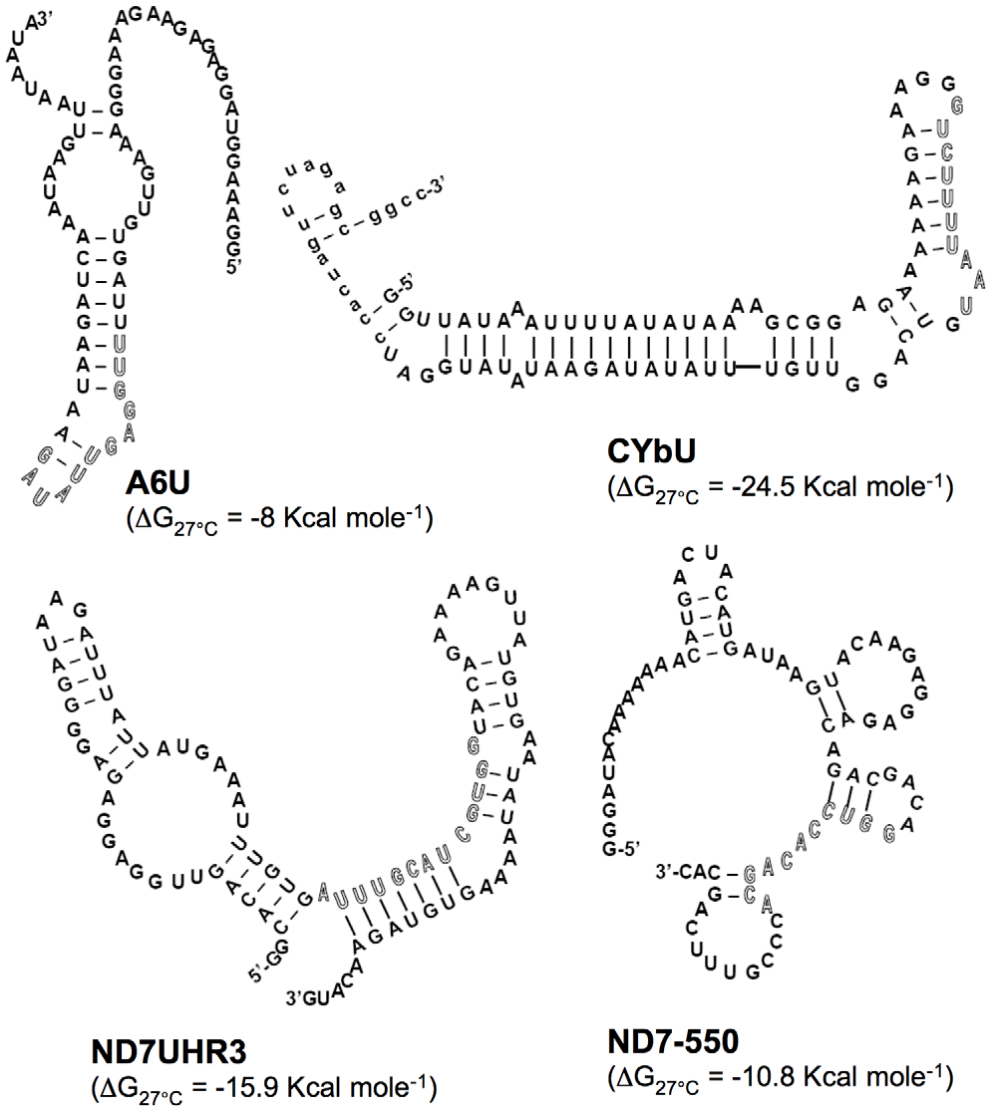
Predicted secondary structures for A6U, CYbU, ND7UHR3 and ND7-550. The anchor binding site is represented in outline font.

Computer modeling of the mRNAs indicate that they have distinct secondary structures around the ABS (Fig. 2). A6U forms the least stable structure and presents most of the anchor binding site within a terminal loop, defined by an 8 bp stem (ΔG_27°C_ = −8 Kcal mole^−1^). In contrast, CYbU forms a stable stem-loop with its ABS located mostly in a double-stranded region within the stem (ΔG_27°C_ = −24.5 Kcal mole^−1^). The structures obtained by enzymatic and chemical solution structure probing of A6U and CYbU support the computer predicted structures [22], [23]. Both ND7 mRNA substrates had predicted structures that were less stable than CYbU (ND7UHR3, ΔG_27°C_ = −15.9 kcal mole^−1^ and ND7-550, ΔG_27°C_ = −10.8 kcal mole^−1^). ND7UHR3 was predicted to have the ABS within a double-stranded region with internal loops, while the ABS for ND7-550 was mainly in a single-stranded region. In addition, the ND7-550 target sequence is unusual in that it is very C-rich.

### Determination of ABS accessibility

**Figure 3:**
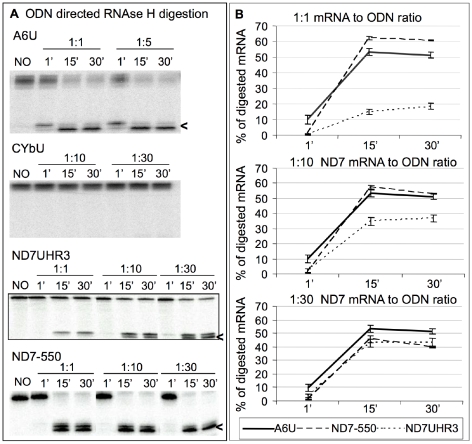
ODN-directed accessibility assays. **A**. Representative images of 8% denaturing polyacrylamide gels. Each reaction contained a pre-hybridized ^32^P-labeled mRNA (A6U, CYbU, ND7UHR3, or ND7-550) that was digested with RNase H for 1, 15, and 30 minutes upon addition of a specific ODN (1∶1, 1∶5, 1∶10, or 1∶30 mRNA to ODN ratio). “NO”: no ODN control. The digested products (<) are indicated. **B**. Percentage of RNase H digestion products. For comparison purposes, the amount of digested A6U shown in each graph was kept constant at 1∶1 ratio. The CYbU mRNA was not included because no digested products were detected. These data are the average of three experiments.

To confirm the predicted ABS accessibility, we conducted RNase H-mediated cleavage assays [24], [25]. In these experiments, 10 pmols of 5′-^32^P-labeled mRNA were renatured *in vitro* (40 mM Tris-HCl pH 7.5, 100 mM KCl, 2 mM MgCl_2_, 1 mM DTT). RNase H and a 10–11 nt oligodeoxyribonucleotide (ODN) complementary to the ABS were concomitantly added to the folded mRNA and aliquots were taken for analysis. The relative accessibility of the target sequence was evaluated by using different ratios of mRNA to ODN, including 1∶1, 1∶5, 1∶10, and 1∶30 ratios, and by assaying for cleavage at three different time points (1, 15, and 30 minutes). The reactions were specific and demonstrated reproducible and expected cleavage products (Fig. 3A). Quantitative analyses of substrate cleavage revealed different degrees of digestion, depending on the mRNA, the incubation time, and the ODN concentration (Fig. 3B). The A6U cleavage at the lowest (1∶1) ratio is shown in all three panels for comparison purposes. As predicted, the two substrates with the ABS within a single-stranded region (A6U and ND7-550) were the most accessible, showing the highest ODN-directed cleavage (Fig. 3B). In contrast, the CYbU substrate showed no cleavage at even the highest ODN concentration, indicating the energetic difficulty involved in invading the stable mRNA stem-loop structure. Cleavage of the ND7UHR3 substrate was observed at the lowest (1∶1) ratios and the percentage of digested ND7UHR3 did increase with increasing amounts of ODN and time of digestion. Nevertheless, its maximum digestion was substantially lower than the percentage of A6U cleaved at the lower mRNA to ODN ratios (Fig. 3B). These results suggest that this assay can be used to quickly assess the accessibility of specific gRNA targets.

### Analysis of equilibrium binding affinities

**Figure 4:**
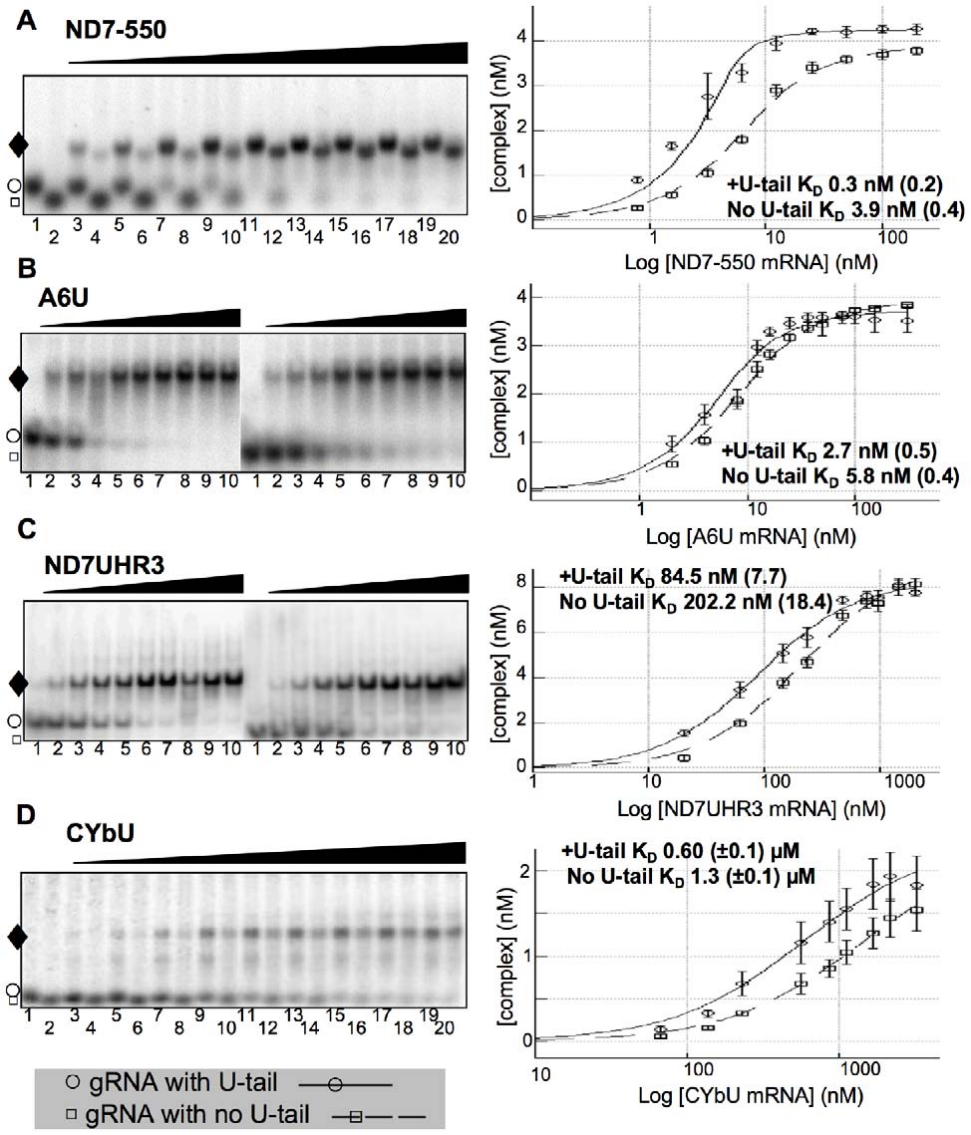
mRNA/gRNA binding affinity by EMSA. **A–D**: Representative images of 6% polyacrylamide gels and corresponding binding isotherms with the apparent dissociation constants (K_D_). Samples contained 5 nM ^32^P-labeled gRNA (gA6-14, gCYb-558 and gND7-550) or 10 nM ^32^P-labeled gRNA (gND7-506) and increasing concentrations of the cognate mRNA. For the ND7-550 (**A**) and CYbU (**D**) gels, the odd number lanes are for gRNA containing its U-tail (○) and even number lanes for the gRNA with deleted U-tail (□). For the A6U (**B**) and ND7UHR3 (**C**), the gRNA +U-tail and with no U-tail were separated. For mRNA concentrations used, see materials and methods. Complex formation (♦) was quantified and the K_D_ calculated as described in the materials and methods. The binding isotherms show the average result obtained from 4 experiments for each pair of mRNA/gRNA. Error bars indicate the standard deviation in complex formation. The calculated error in K_D_ is shown in parentheses.

To correlate ABS accessibility and sequence with efficiency of RNA duplex formation we determined the binding affinity for the mRNA/gRNA pairs using EMSA, as previously described [13]. In these experiments, ^32^P-labeled gRNA, either with or without its U-tail, was annealed with increasing concentrations of mRNA in a buffer containing 2 mM Mg^++^. The cognate RNAs were combined, denatured at 70°C for 2 min, and then allowed to anneal for 3 hours. Free RNAs were separated from the bound complex by electrophoresis on nondenaturing 6% polyacrylamide gels. For all gRNA/mRNA pairs a single predominant band was observed (Fig. 4). Complex formation was quantified on Molecular Dynamics phosphorimager and the observed dissociation equilibrium constant (K_D_) calculated. Surprisingly, the observed K_D_ for the ND7-550 pair was almost 10 fold lower than the K_D_ measured for the A6 pair (K_D-ND7-550_ = 0.3±0.2 nM vs K_D-A6U_ = 2.7±0.5 nM). As predicted, the binding affinity for the ND7UHR3 was weaker, K_D_ = 84.5±7.7 nM, but still considerably better than CYb. In addition, for all gRNA/mRNA pairs, the U-tail increased the affinity of the gRNA for its cognate mRNA. The U-tail contribution was minimal for the A6U/gA6-14 interaction, decreasing the observed K_D_ approximately 2-fold. In contrast, a difference in binding affinity of over 10-fold was observed in the presence of the U-tail for the ND7-550 pair. This indicates that the gRNA U-tail can significantly contribute to the binding affinity even for those gRNAs that show high affinity for their targets.

### mRNA/gRNA rate constants

**Figure 5:**
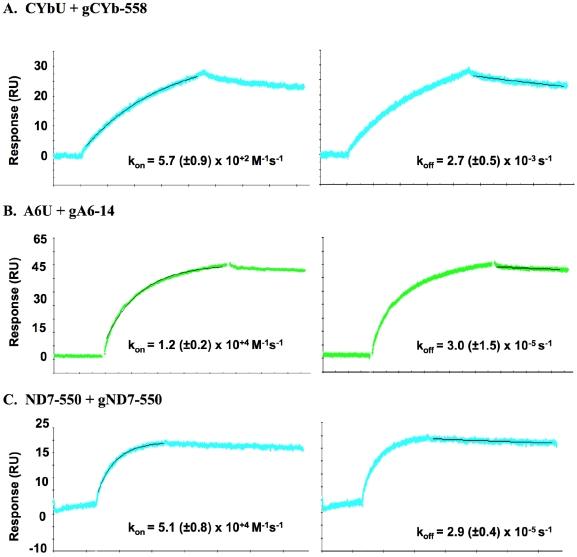
mRNA/gRNA rate constants by Surface Plasmon Resonance. Representative SPR sensograms are shown with line fits. **A**. CYbU+gCYb-558. **B**. A6U+gA6-14. **C**. ND7-550+gND7-550. The association (k_on_) and dissociation (k_off_) rate constants represent the mean of a minimum of 3 runs (each run utilizing 3 different mRNA concentrations) and are listed with the error in parentheses. RU = resonance units.

The efficacy of RNA-dependent systems has been correlated to fast annealing kinetics [26]. To define the association (k_on_) and dissociation (k_off_) rate constants of the mRNA/gRNA interaction, surface plasmon resonance (SPR) was employed [27]. In these experiments, an ODN-tag with a 3′ biotin label was ligated to the 3′-ends of the target mRNAs using T4 DNA ligase and a bridge ODN. The biotin labeled mRNA was then immobilized to the streptavidin covered surface of the SA chip. To see reliable gRNA binding to the mRNA, 50 to 150 RU of mRNA were attached to the chip surface, in two of the four channels. One channel remained empty to be used as a reference and one contained only the biotinylated tag as control for background binding. A continuous flow of gRNA in binding buffer (100 mM Tris pH 7.5, 0.1 mM EDTA, 2 mM MgCl_2_, and 100 mM KCl) was injected over the immobilized mRNAs to monitor association. The dissociation phase was obtained by chasing the gRNA with buffer for up to three hours. The long injection times and the regeneration procedures used between two binding assays progressively affect the mRNA integrity during the experiments [28]. These limitations made it difficult to generate curves amenable to simple Scatchard-type analyses. Because line fitting using global analysis requires extremely high quality data, this method of analysis proved impractical [29]. However, using the separate fit function of Biaevaluation 3.0 (Biacore, Uppsala, Sweden) for each association and dissociation curve separately, allowed an analysis of the individual rate constants. The individual rate constants were averaged from a minimum of three separate experiments (three mRNA concentrations per experiment), and the equilibrium dissociation constant was calculated from the rate constants. The errors reported are based on the variances of all curves obtained [30]. Figure 5 shows representative binding curves for the CYbU, A6U and the ND7-550 interactions. For both A6U and ND7-550, the SPR analyses indicate that the gRNA/mRNA interactions are very stable with a very slow dissociation rate of ∼3.0×10^−5^ s^−1^. In contrast, the association rates differed significantly, with the gND7-550 gRNA binding its target ∼4 fold faster than gA6-14 (5.1×10^4^ M^−1^s^−1^ vs 1.2×10^4^ M^−1^s^−1^). Using the measured rate constants, the affinity constants (K_D_) for both RNA pairs were calculated to be 2.5 nM and 0.56 nM for the A6U and ND7-550 pairs, respectively. The calculated K_D_s were very similar to the K_D_s observed by EMSA, indicating that increase in affinity observed for the ND7 pair was due to the difference in association rate.

In contrast, the gCYb-558 association rate with CYbU was very slow (5.7×10^2^M^−1^s^−1^) and its dissociation rate significantly faster (2.7×10^−3^M^−1^s^−1^). These rate constants probably reflect the difficulty in displacing the stable stem-loop structure formed by the 5′ end of the CYbU transcript. This suggests that this complex rarely forms *in vivo* without help from an annealing factor, allowing for the observed regulated editing of CYb during the complex life cycle.

### Ability of each mRNA/gRNA pair to be recognized and cleaved by the editosome

**Figure 6:**
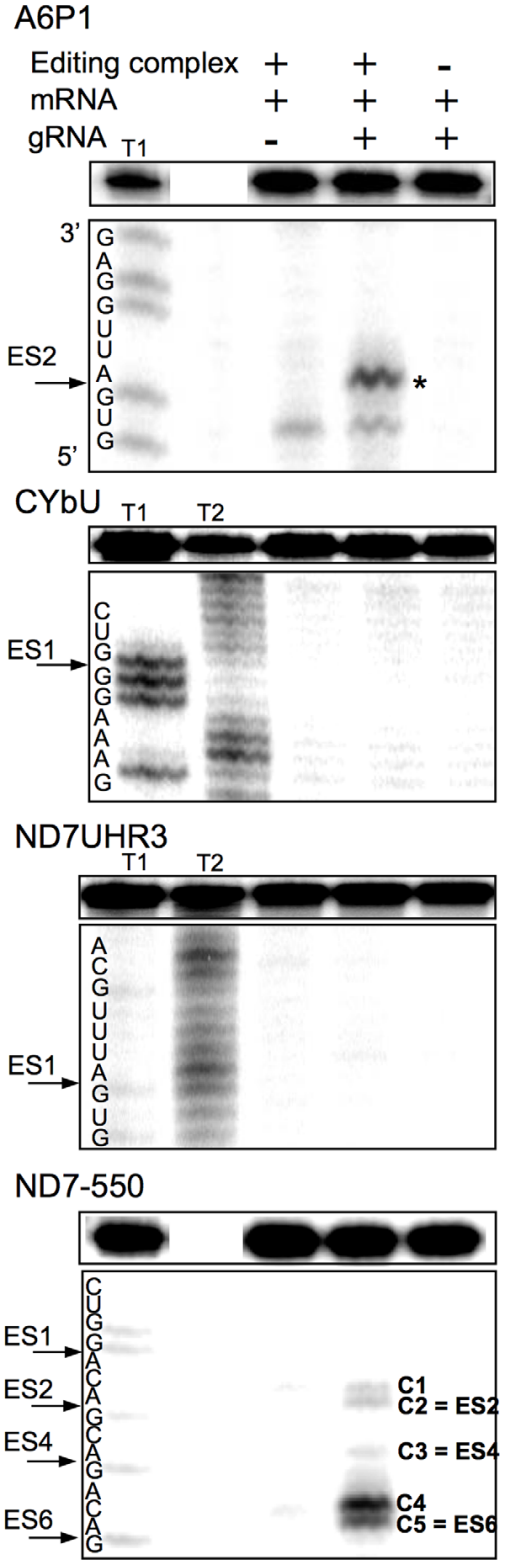
*In vitro* gRNA-directed cleavage assay. The panels are representative images of 8% denaturing polyacrylamide gels, where the top boxes contain the input mRNA. Radiolabeled mRNAs (A6P1, CYbU, ND7UHR3, ND7-550) were incubated with their cognate gRNAs (gA6-14, gCYb-558, gND7-506, and gND7-550) in standard cleavage conditions with column purified editosomes. The asterisk (*) indicates the gA6-14 directed cleavage product at the correct editing site. gND7-550 directed cleavages are indicated (C1–C5). C1 and C4 cleavages map to sites that are normally not edited in the mature transcript. C2, C3 and C5 map to predicted editing sites ES2–6, as indicated. Cleavage products migrate ½ nucleotide slower than the products generated with RNase T1 digestion due to differences in enzyme site of cleavage. T1 and T2: RNase T1 and T2 digests.

**Figure 7:**
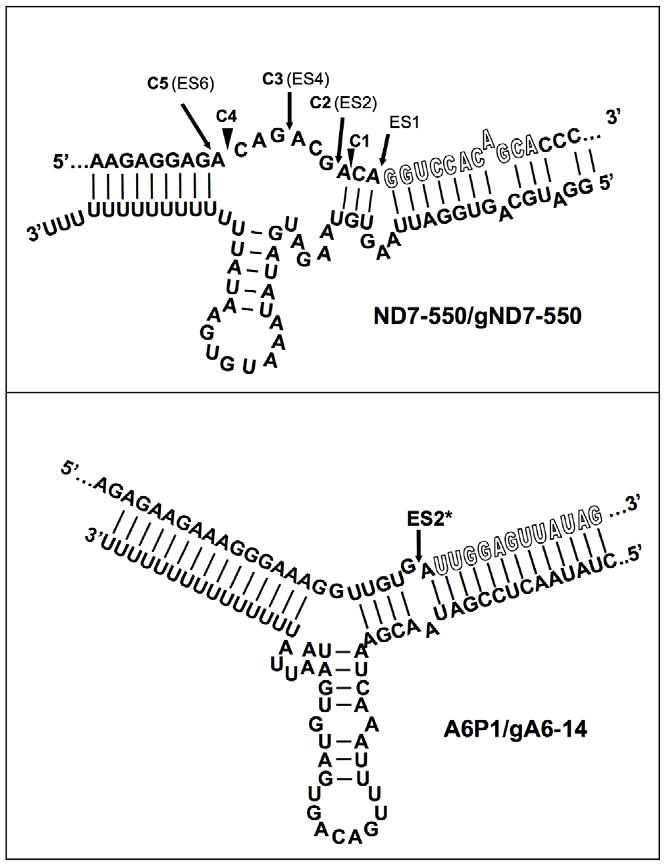
Predicted secondary structures for the A6P1/gA6-14 and ND7-550/gND7-550 complexes. Sites where we observed gRNA-directed cleavages C1–C5, are indicated. ES = Editing Site. ES2* indicates the first editing site that is correctly cleaved in the A6P1/gA6-14 interaction. No cleavage at at the first editing site was observed for ND7-550. The gRNA-dependent cleavages for this substrate occurred at sites that are edited in the mature transcript (C2 - ES2, C3 - ES4 and C5 - ES6) and at sites that are not edited in the mature transcript (C1 and C4). The mRNA anchor binding sequence is shown in outline font.

**Figure 8:**
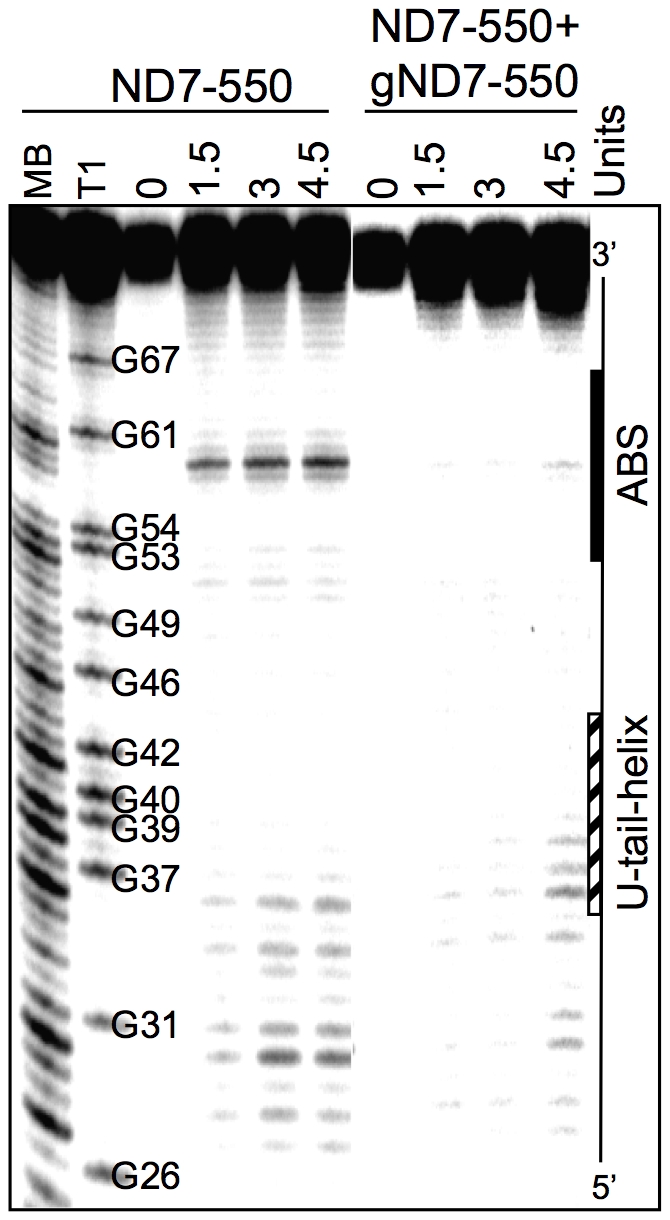
Solution Structure Probing of ND7-550/gND7-550. Representative image of a denaturing polyacrylamide gel. The ND7-550 mRNA was 5′-end-labeled and renatured alone or with gND7-550. 0: no enzyme control. 1.5, 3, and 4.5 Units: amount of Mung Bean nuclease. MB and T1 are Mung Bean and RNase T1 digests for sequence mapping. The position of the ABS and the region where the gRNA U-tail is predicted to bind on the mRNA are indicated.

Using standard editing reaction conditions, we evaluated the ability of the mRNA/gRNA pairs used in this study to undergo cleavage in a gRNA-directed cleavage reaction. The assay consisted of incubating the 5′-^32^P-labeled mRNAs with the respective cognate gRNAs in the presence of mitochondrial extract containing the editing machinery. To standardize the reactions to be of the insertion type, the A6 mRNA was partially edited at its first site (A6P1, figure 1). All four mRNA/gRNA pairs, in a molar ratio of 1∶10, were pre-hybridized and incubated with the above mitochondrial extracts for 1 h, at 27°C. Under these conditions, gRNA-dependent cleavages at editing sites were observed only in A6P1 and ND7-550 (Fig. 6). As expected, A6P1 was cleaved at the first expected site (ES2*) in a gRNA-directed manner. A second cleavage product was observed, however it was also present in the absence of the gRNA indicating the presence of a nonspecific endonuclease. The cleavages within ND7-550 were also gRNA dependent (note absence of cleavage product when exogenous gRNA was not added). However, five different cleavage sites, C1–5, were observed, none of them at the predicted first editing site (ES1) (Fig. 6 and 7). C2, C3 and C5 did coincide with known editing sites (ES) 2,4 and 6, respectively. C1 and C4 mapped to sites that are normally not edited in generation of the mature transcript. C1, 2, and 3 occurred with varied efficiencies between independent assays (data not shown). The C4 and C5 cleavages however, were reproducible and almost as efficient as the cleavage observed at ES2 in the A6 mRNA (efficiencies of approximately 3% (C4) and 1.8% (C5) in ND7-550 and 4% in the A6). Experiments using either enriched glycerol fractions or column-purified editosomes gave very similar results (data not shown). Computer modeling of ND7-550/gND7-550 indicates that the cleavages occurred within a single-stranded region that is punctuated with three cytosine residues located just upstream of the anchor duplex. The U-tail is predicted to base pair farther upstream with an uninterrupted run of 9 purines (Fig. 7). To insure that the observed cleavages were not due to non-specific single strand RNases present in the mitochondrial preparations, gND7-550 was annealed to ND7-550 in NE buffer 2 at 27°C and the complex's structure was probed with Mung Bean Nuclease (MBN). No digestion by the single stranded specific MBN was observed within the C1–5 region (Fig. 8).

## Discussion

In recent years, it has become clear that the ability of small RNAs to accurately and efficiently pair with specific RNA targets plays a critical role in gene regulation. In both prokaryotes and eukaryotes, small RNAs act post-transcriptionally, regulating a myriad of important cellular pathways (for reviews see refs. [31]–[35]). In *T. brucei*, hundreds of gRNAs are predicted to be involved in generating mature, translatable transcripts in the mitochondrion. Despite the crucial role that gRNAs play in the editing process, very little is known about how the gRNAs specifically and efficiently target their cognate mRNAs. Extensive work has been conducted to characterize the base composition of editing sites. Mutations, deletions, substitutions, and even a more detailed selection-amplification technique have been applied to regions flanking editing sites to investigate what sequences or structural motifs define such specific regions [36]–[39]. However, while these experiments have defined determinants that “enhance” the efficiency of the *in vitro* editing reaction, it is unclear if the introduced changes affect protein recognition sites, or simply the ability of the gRNA to target and effectively pair with the selected editing substrate. A number of accessory proteins have been identified that facilitate the annealing of gRNAs to their targets [40]–[44]. The two most characterized, the MRP1/MRP2 complex and RBP16, are both gRNA binding proteins that appear to play multiple roles within the mitochondrion [45], [46]. Crystal structures of the MRP1/MRP2 bound to a gRNA indicate that it stabilizes the gRNA anchor region in an unfolded conformation, increasing its ability to pair with its target [43]. In contrast, RBP16, with both a cold shock domain and an arginine/glycine rich (RGG) domain, has been demonstrated to have RNA annealing and RNA unwinding activities [44]. Knockout and knockdown studies indicate that these proteins have transcript-specific roles with differential effects on both editing and mRNA stability [45]–[46]. The differential effects of down regulation on RNA editing are particularly enigmatic. For example, while down-regulation of MRP1/2 decreased the levels of edited CYb and RPS12, no affects on editing were observed for a number of transcripts including COII and A6. Intriguingly, down-regulation of MRP1/2 resulted in an increase in editing of the ND7 mRNA. These data, along with the large number of gRNAs required for the editing process, suggests that additional target specific factors are probably required for efficient editing.

The results of this work clearly show the impact of both mRNA structure and target sequence on the ability of gRNAs to recognize and pair with their target mRNAs. For the CYb transcript, the ABS target for gCYb-558 is sequestered within a very stable stem loop structure. Efficient interaction would require a helicase, introducing a step that allows for the regulation of editing of this transcript. In contrast, through EMSA and SPR studies we found very high affinities coupled with fast association for the A6U and ND7-550 pairs. The difference in affinity observed between the ND7-550/gND7-550 interaction and the A6U/gA6-14 interaction (K_D_ of ∼0.3 nM for ND7-550/gND7-550 and ∼2.6 nM for A6U/gA6-14) was due to a four-fold faster association rate constant for gND7-550. This was initially surprising as we observed a much faster RNase H ODN directed cleavage of the A6 mRNA in the accessibility assays. This may be explained by the fact that the ODN is a short piece of DNA and interaction with its target may not require the intermolecular rearrangements necessary for the larger gRNA interaction. The difference in the A6 and ND7-550 SPR-measured association rate could also be correlated with the G-C content of both anchor-binding sites. The ND7-550 ABS contains 7 G-C pairs versus only 4 in the A6 (Fig. 1). The rate-limiting step in most RNA/RNA interactions is an initial base-pairing interaction that forms in a concentration dependent second-order process. The large number of possible G-C pairs may allow a more stable nucleus to form, increasing the probability of the initial interaction continuing on to helix formation.

Only the two most accessible mRNAs, A6 and ND7-550, were cleaved by a gRNA-dependent endonuclease activity present in the mitochondrial extract. However, in contrast to the A6 mRNA, which was specifically cleaved one nucleotide upstream of the anchor duplex, the ND7-550 was cleaved at multiple upstream editing sites. Cleavages upstream of the first editing site have been previously reported to happen *in vitro* and *in vivo*, and are of unknown causes [47]–[49]. Adler and Hajduk [49] suggest that multiple cleavages upstream of the expected ES could involve improper assembly of the exogenous mRNA/gRNA with the purified editing complexes. More recent studies found that the structure surrounding the ES was a strong determinant of association and cleavage by purified editing complexes [50], [51]. The ND7-550 substrate differs from A6 in that it has several C-residues located within the first 10 nt 5′ of the ABS (Fig. 8). Thus, the U-tail interaction is predicted to occur further upstream, increasing the number of nucleotides flanked by the anchor and U-tail helices [23]. This might limit the gRNA's ability to direct editing to the 3′ most-site *in vitro*. It may be that while A6 can present the correct editing site to the editosome in the absence of additional proteins (proteins not found in the core editosome), other substrates, like ND7-550, may need additional accessory factors for proper folding and correct presentation. In contrast to both A6 and ND7-550, the two substrates with anchor-binding sites found within highly structured region were not able to either efficiently pair with their gRNAs or assemble with the core editosome. These targets probably require accessory proteins for efficient gRNA pairing.

In summary, the results of these experiments indicate that target structure and sequence can significantly affect the ability of the gRNA to effectively target a substrate for RNA editing. The variety of secondary and tertiary features that must be disrupted for gRNAs to bind suggests that different gRNA/mRNA pairs might require different “sets” of accessory factors for efficient editing. By dissecting the requirements for effective RNA targeting and hybridization, we can obtain an improved understanding of the requirements for functional interactions between small RNA and their targets.
